# “Part of the Team”: Mapping the outcomes of training patients for new roles in health research and planning

**DOI:** 10.1111/hex.12591

**Published:** 2017-06-28

**Authors:** Svetlana Shklarov, Deborah A. Marshall, Tracy Wasylak, Nancy J. Marlett

**Affiliations:** ^1^ Patient and Community Engagement Research (PaCER) Program O’Brien Institute for Public Health University of Calgary Calgary AB Canada; ^2^ Community Rehabilitation and Disability Studies Program Cumming School of Medicine University of Calgary Calgary AB Canada; ^3^ Department of Community Health Sciences Cumming School of Medicine O'Brien Institute for Public Health University of Calgary Calgary AB Canada; ^4^ Strategic Clinical Networks Alberta Health Services Calgary AB Canada; ^5^ Cumming School of Medicine University of Calgary Calgary AB Canada

**Keywords:** capacity building, chronic disease management, health culture, patient engagement, patient roles, peer‐to‐peer research

## Abstract

**Background:**

A patient research internship (Patient and Community Engagement Research program—PaCER) was created to support a provincial commitment by Alberta Health Services’ Strategic Clinical Networks^™^ to find new ways to engage patients in a new interdisciplinary organization to support evidence‐informed improvements in clinical outcomes across the health system.

**Objective:**

Implement and test a new research method and training curriculum to build patient capacity for engagement in health through peer‐to‐peer research.

**Design:**

Programme evaluation using Outcome Mapping and the grounded theory method.

**Setting and Participants:**

Twenty‐one patients with various chronic conditions completed one year of training in adapted qualitative research methods, including an internship where they designed and conducted five peer‐to‐peer inquiries into a range of health experiences.

**Main Outcome Measures:**

Outcomes were continually monitored and evaluated using an Outcome Mapping framework, in combination with grounded theory analysis, based on data from focus groups, observation, documentation review and semi‐structured interviews (21 patient researchers, 15 professional collaborators).

**Results:**

Key stakeholders indicated the increased capacity of patients to engage in health‐care research and planning, and the introduction and acceptance of new, collaborative roles for patients in health research. The uptake of new patient roles in health‐care planning began to impact attitudes and practices.

**Conclusions:**

Patient researchers become “part of the team” through cultural and relationship changes that occur in two convergent directions: (i) building the capacity of patients to engage confidently in a dialogue with clinicians and decision makers, and (ii) increasing the readiness for patient engagement uptake within targeted organizations.

## 1 INTRODUCTION

Involving patients as stakeholders in health‐care decision making can benefit the effectiveness and sustainability of services^1^, ^2^, ^3^ and is one of the central components of health‐care policy development.^4^, ^5^ Training patients in new methods of engagement and research has enabled a shift from traditional, professionally directed health‐care delivery towards a greater inclusion of patients and community networks.^6^ A variety of patient or user initiatives related to health research have been introduced internationally and are based on a rich tradition.^7^ Some prominent examples are INVOLVE,^8^ the Patient‐Centered Outcome Research Institute,^9^ and the Service User Research Enterprise (SURE).^10^ Engaging patients in research has great potential for empowering them in “doing and sharing research that deals with their well‐being,”^11^ which, in turn, can catalyze their competence, self‐confidence and engagement in negotiating with health‐care policy change and research.^12^, ^13^

This international and domestic trend^14^ enabled Alberta Health Services (AHS), the first province‐wide health system in Canada, to create innovative approaches to sustainable health‐care delivery, prevention and wellness for the entire province using an innovative approach to engaging patients and community members. The Strategic Clinical Networks^™^ (SCNs)^15^, ^16^ are the mechanism to promote evidence‐based, clinician‐led, team‐delivered health improvement strategies and innovation across the province.^17^ Funding received through a grant, “Patients Matter: Engaging Patients as Collaborators to Improve Osteoarthritis (OA) Care in Alberta” (2011‐2013), enabled the planners of this health‐care restructuring initiative to engage patients as partners in the SCNs.^18^ The task of changing citizen engagement in health‐care decision making required an investment in building the capacity of patients and family members for using new engagement research skills. It also required efforts within AHS, and specifically the SCNs, to make sure that the patients’ skills were utilized, and their newly trained voice heard before any potential shift in culture could occur. Citizens living with various chronic conditions were recruited to be part of an internship training programme to test this innovative approach and evaluate the outcomes.^1^

The objectives of our project were as follows: (i) build the capacity of patients to engage confidently with clinicians and decision makers through their engagement research expertise;^19^, ^20^, ^21^ (ii) bring a positive change to the culture and systemic factors that could drive patient engagement in health care;^3^ (iii) promote the readiness for patient engagement uptake within the targeted organizations.^22^ This 2‐year study introduced a radical shift in roles of patients in health care that may encourage training for patients to take up other collaborative roles in health, research and decision making.

## 2 MATERIALS AND METHODS

### 2.1 Framework for evaluation

Outcomes were continually monitored and evaluated using an Outcome Mapping framework.^23^ Outcome Mapping was chosen as a particularly relevant framework for programme evaluation because it begins at the point of designing the intervention and then continuously monitors and evaluates implementation, changing directions with input from all participating partners. Outcome Mapping, as a methodology for tracking social change using sound research methods of data collection and decision making, was recommended by the Canadian Foundation of Healthcare Improvement, the national funder of this initiative. We used Outcome Mapping to test the effectiveness and impact of investing in patient training to develop new social roles as patient engagement researchers. Outcome Mapping addresses the organizational and social ecological factors of change.

To augment and deepen our outcome analysis, we used the grounded theory analytical method that enabled us to focus on the changes experienced by the participating patients and SCNs.^18^, ^24^, ^25^, ^26^ Through this combination of the Outcome Mapping design and monitoring with the grounded theory analytical approach, we conducted regular and consistent data collection from all stakeholders, including formal interviews, data capture of meetings and workshops within real‐world settings of the internship training, as well as within the interactions with the SCNs.

The Outcome Mapping method was developed originally by the International Development Research Centre based on Canada's work in South America, Africa, Asia, India in consultation with the American Pacific Institute for Research and Evaluation who provided their experience with social services, health and community development. This method is used widely in Canada and other countries to provide systematic mapping and standardized data collection methods for tracking and reporting projects and programmes that include complex social networks and large‐scale change. Outcome Mapping not only tracks predicted outcomes but uses a collaborative process of stakeholder involvement throughout the change process to maximize the sustainability and build capacity of stakeholders.

### 2.2 Intervention

The investment in building patient capacity included the adaptation of a citizen‐led research curriculum^27^ and the development of a year‐long internship for patients and family members by a faculty member from the University of Calgary. The internship was titled Patient and Community Engagement Research (PaCER), where citizens with a variety of health conditions learned both engagement and research skills. It was hypothesized that patients who were “trained” in unique skills that engaged other patients could add an important patient research perspective to the core committees of each of the SCNs, whose mandate is to improve care and clinical outcomes, advance research and accelerate knowledge translation. These trained PaCERs represent not only their own experience, but the collective experience of other patients acquired through their research.

The internship included competence‐based training in four relevant qualitative research methods: (i) participant observation (fieldwork);^28^ (ii) interviews and questionnaires including structured and open‐ended options;^29^ (iii) focus groups^30^ and (iv) narrative interviewing.^31^, ^32^ The training consisted of 120 hours of classroom activity focused on specific adapted methods of qualitative inquiry built from previously published work.^27^ The internship also included designing and conducting group research studies, in which PaCER interns applied their newly acquired research skills and used specifically designed methods to engage patients, families and communities (Data S1). Each PaCER intern group of four to eight patients designed a study and wrote a proposal that was submitted for approval to both the University of Calgary Conjoint Health Research Ethics Board and the relevant SCN core committees. This also laid a foundation to engage the SCN leadership and facilitate uptake of the findings into their health planning work and transformational roadmaps. Upon approval, intern groups conducted their studies and reported their findings (Table 1).

**Table 1:** The five research studies designed and completed by PaCER interns during the Outcome Mapping study

1. *The experience of living with chronic joint pain* A qualitative study exploring the experience of patients living with chronic joint pain, whether undiagnosed or diagnosed as osteoarthritis, for which there was no cure, with the purpose to bring the reality of these patients to the attention of both the medical and general populations
2. *The experience of waiting for help with osteoarthritis* A study aiming to bring the patient perspective to the understanding of what it means for patients with osteoarthritis to wait for health care, encountering uncertainty and loss of control in waiting for medical events to unfold
3. *Oh! Canada: Southeast Asian immigrants’ experience of osteoarthritis surgery* A narrative study conducted by a multi‐lingual group of PaCER interns, focusing on the experiences of people with osteoarthritis—members of the Southeast Asian Community—who had reached “the end of the road” and had to have joint replacement surgery
4. *The hidden pathways of chronic illness* A study conducted by PaCER interns with a variety of health conditions, exploring how patients make sense of living through chronic illness. This study concentrated on patient experiences of finding and making pathways through their chronic illness *apart* from their journey through the health‐care system and the clinical pathways
5. *A case study of engagement at Wellspring Calgary: What works and how?* This study, built upon the theory of salutogenesis and based on the participant observation method, was undertaken by a team of six PaCER interns who had experience of cancer and decided to embark on a case study to explore the experience of attending a community‐based cancer support and wellness centre (Wellspring Calgary), and to understand how such an organization might influence members to become more engaged in their health and wellness.
Publishing Status by late 2013
All five studies were delivered by patient researchers in local, national and international scholarly seminars and conferences (11 presentations and posters in Canada, US, and Berlin)
At least 21 invited presentations locally for community audiences by the end of 2013
*Challenges*: Publishing was one of the desirable outcomes, and challenges in scholarly publication were expected, considering that these were non‐traditional patient‐led, student studies. While authoring their publications upon graduation, PaCERs encountered time constrains as they became engaged in many SCN activities. Currently, publications are underway: one study is under peer review with an academic journal (authored by patient researchers), and others are being prepared for publication with the University of Calgary Press

PaCER, patient and community engagement research.

The SCN leadership was part of the team that developed the curriculum and mentored the research work of the PaCER interns. Several members of the SCNs provided mentorship and stewardship for the research inquiry and acted as field mentors for the research. The inclusion of SCN leadership, health scientists and faculty members in the implementation of the internship provided a unique opportunity to challenge cultural expectations about patient engagement and facilitate changes in their own domains and in health system planning.

### 2.3 Study participants

Outcome Mapping defines groups, organizations and individuals directly involved in the study of change as “boundary partners”.^23^ We aimed at engaging all participating partners in the design, monitoring and evaluation of our project. Boundary partners are active agents within the initiative who benefit and change as a result of its implementation. They continue sustainable change once the programme is finished. Our boundary partners were: (i) PaCER interns (21 participants)—patients with various health conditions, including some family members, recruited for this training programme through clinics, forums, posters, information sessions, media and newsletters and word of mouth; (ii) the AHS Bone and Joint Health SCN; and (iii) the University of Calgary's O'Brien Institute for Public Health. The AHS and academic stakeholders (15 key informants) were recruited through the emerging SCNs.

Two cohorts of patients who volunteered as PaCER interns for the training were recruited to the project. Our recruitment criteria were inclusive, inviting adults with patient experiences who were willing to commit to completing the programme. The first cohort invited patients with osteoarthritis, and the second cohort included patients regardless of their specific diagnosis. No additional selection procedures were required (e.g, we did not require police checks). Participants were reimbursed for their travel and parking, and food was offered on training days. Training was provided free of charge. We did not screen potential participants—rather, they self‐selected once they started, and were free to stay or withdraw at any point in the study. In total, of the 46 recruits (two cohorts within two years), 21 interns graduated, including nine patients with osteoarthritis and 12 patients with other health conditions (e.g, cancer, mental illness, cardiovascular, kidney conditions and diabetes). The group was diverse by education level (from high school or Bachelors level to PhD), cultural background (immigrant experience, homelessness experience, seniors), employment (employed full‐ or part‐time, receiving disability benefits or retired), age (from late 30s to 75) and gender (17 women and four men).

### 2.4 Data collection and analysis

Outcomes Mapping is built on three stages. In the first stage, Intentional Design, all partners create a vision of desired outcomes and strategies to bring about the changes—the “Outcome Challenges” (Tables 2, 3, 4 and Table S1). In the second stage, Outcome and Performance Monitoring, on‐going progress monitoring tracks the changes identified in stage one. At the third stage, evaluation priorities are identified and the evaluation findings are compared against the outcomes initially envisioned by partners.

**Table 2:** Patient engagement researchers: envisioned outcomes

Boundary partner 1	Outcome challenge 1
Patient engagement researchers—project participants	The programme intends to see the group of project participants who are skilled and active as patient engagement researchers. They have mastered the specific research skills and know how to engage other patients, capture and articulate their ideas, support these ideas with valid research and bring them to the table. Patient participants understand, value and are able to act in their new roles as patient engagement researchers. They are becoming mentors to other patients who are interested to also take up these roles. They begin to participate in the Bone and Joint Health Strategic Clinical Network as knowledgeable, competent and assertive partners in decision making

**Table 3:** Bone and Joint Health Strategic Clinical Network: envisioned outcomes

Boundary partner 2	Outcome challenge 2
Bone and Joint Health Strategic Clinical Network, Alberta Health Services	The programme intends to see the Bone and Joint Health Strategic Clinical Network that will deploy patient researchers as members on their working groups and committees as equal partners. In the longer term, the Alberta Health Services will adopt and use the model of patient engagement research in the Bone and Joint Health Strategic Clinical Network as a prototype for the development of the other Strategic Clinical Networks (for example, Addictions and Mental Health, Cardiac or Cancer Networks)

**Table 4:** The O'Brien Institute for Public Health: envisioned outcomes

Boundary partner 3	Outcome challenge 3
O'Brien Institute for Public Health, Cumming School of Medicine, University of Calgary	The programme intends to see the O'Brien Institute for Public Health that will sustain and support future patient engagement research opportunities through multidisciplinary faculty and students, as well as linkages with other boundary partners such as Alberta Health Services

Data collection in our study included tools provided within the Outcome Mapping method, for example, an outcome journal and strategy journal, augmented by participant observation throughout all project activities and team meetings. We conducted regular (at least bi‐monthly) monitoring meetings with all boundary partners (partner organizations), with clear expectation that project strategies would be continually adjusted and improved based on the emerging data flow. In addition, we conducted two sets of semi‐structured face‐to‐face interviews and surveys in the beginning of the project, and at the end of the project, we conducted focus groups with participants and documentation reviews (Table S2).

Our main objective was to implement and test a new research method and training curriculum to build patient capacity for engagement. We used Outcome Mapping as a collaborative design tool. As the project progressed, the emerging data led us to a deeper theoretical investigation, as our observations started to show a potential for changing relationships between patients and medical professionals, decision makers and researchers. We were interested in studying these emerging processes. The grounded theory analysis method^25^, ^26^ was particularly effective for this purpose, including coding techniques at various levels of conceptualization and comparison, leading to the emergence of increasingly abstract categories that described the experiences of patients and the development of a theoretical evaluation of the outcomes.

## 3 RESULTS

Twenty‐one PaCERs graduated with competence to conduct collaborative research. As part of their internship, PaCERs completed five studies (Table 1). Tables 2, 3, 4 present the Outcome Challenges designed at the start of the initiative. The most important set of tangible outcomes were associated with training and research completed, as well as with the graduates’ immediate and continuing engagement with the SCNs and academic research teams (Table 5).

**Table 5:** Indicators of tangible results of the outcomes mapping evaluation by the end of the project (all results are presented according to the final report in 2013)

Indicator	Value and explanation
Number of PaCER interns graduating	21 PaCERs
Number of PaCER skill‐training sessions	38 in‐class full‐day instruction sessions in total
Number of hours of training received by each participant	240 h of training *for each of the two cohorts*:120 h in‐classroom training and an equivalent of 120 h of internship=240 h
Number of PaCER research projects approved by the University of Calgary Conjoint Health Research Ethics Board and SCNs	Five research studies
Number of PaCER research projects completed and reported by interns	Five research studies
Number of patients engaged as PaCER study participants (*not* including PaCER interns)	Participant numbers in five studies respectively: 46+20+16+21+22=125
Number of PaCERs—members of SCN core committees and working groups	Eight PaCERs are members of five Strategic or Operational Clinical Networks (Bone & Joint SCN; Seniors Health SCN; Obesity, Diabetes & Nutrition SCN; Cardiovascular & Stroke SCN; Surgery OCN) by the end of the project
Number of PaCERs invited to consider joining SCN (in addition to the above)	Two PERs invited to join Cancer Care SCN
PaCER internship sustainability	PaCER internship was continued after the project was concluded, sponsored by SCNs and other organizations (beginning in January 2014)
Number of newly planned collaborative research projects to include graduating PaCERs (with SCNs, IPH and other partners)	At least six funded research projects being designed by the end of the project (2013) to build in a PaCER component, hiring six PaCERs as project team members
Number of media and on‐line appearances	11
Number of academic presentations (local seminars, workshops and formal international conferences)	11
Number of invited public and community presentations by PaCERs (eg, presentations to SCNs, community agencies, public groups, Arthritis Society and other)	21

PaCER, patient and community engagement research; PaCERs, patient and community engagement researchers; SCN, Strategic Clinical Network.

Based on these concrete, descriptive outcome data, this analysis follows the logic of grounded theory, conceptualizing the changes observed within the two years of reported data. As the data were analyzed, two major questions emerged from the evidence: What changed for people when they became patient engagement researchers and conducted research? What changed within the health‐care system when “patients” became researchers and got meaningfully engaged?

The emerging core process of patient researchers “becoming part of the team” was conceptualized within the relationship and discourse changes in two convergent directions. First, the patients’ new capacity and role as researchers provided them with confidence, knowledge and a legitimate place at the health‐care decision‐making table. Second, the perceptions within the targeted health‐care organizations were shifting towards acceptance and uptake of this newly informed patient input. In this analysis, we are presenting the growth and challenges within each of these clusters of change.

### 3.1 Patient engagement researchers: “knowledgeable, competent and assertive partners”

Patients’ experiences of change while conducting this research and becoming engaged in the SCNs are captured within the following categories.

#### 3.1.1 The legitimate role at the table

The PaCER training produced skillful qualitative researchers with patient experience. This emerged as a genuine, solid role for patients within health care culture, making their engagement legitimate. This change was achieved intentionally (designed and evaluated through outcome mapping), through establishing partnerships between PaCERs, health science researchers and health‐care providers in joint projects, team collaborations and participation in governance bodies. The emerging PaCER roles extended beyond the conventional perceptions of engaged patients as advisors, advocates or volunteers:

Most patients would come and typically they would have a bad history of something that happened… and they are explaining just from their viewpoint, whereas the PaCERs, with the training and the experience as they go through research… they come and they'll say, Having interviewed a hundred patients, here is what they are telling us. And this is much more powerful, it's not that they are speaking for themselves—they are speaking from much larger body, and this is much more informative than any of the individual providers or researchers have—we just have our own narrow window. [SCN executive].

The impact of PaCER went beyond the specific research results and was felt at the level of relationships within the SCNs and University:

There is more than just research happening. People are invited to be at the table, and people are listened to. Because it's not like they have a white coat on, but they do have ‘A Coat’ on. They don't have a clipboard or a stethoscope, but they got at the table, and people do listen…. Somehow people are willing to listen in a way that had been hard to achieve. [Project team member].

Meaningful patient roles facilitated new conversations with health‐care providers and decision makers and, in the process, improved patient engagement.

#### 3.1.2 Competence and empowerment

Participants gained confidence in their new roles: “I can see myself actually being able to apply the appropriate research techniques which would have been absolutely outside my expertise five months ago”; “I now feel it would be possible to contribute to positive changes in the system.” While the challenges of time and commitment were evident, participants noted feelings of personal gain, enjoyment and being accepted as valuable contributors: “Joining this project has given me a much needed purpose and focus”; “…has given me back a measure of self respect”; “I have felt welcomed.” Participants also demonstrated growth, contribution, increased knowledge and competence:

Well, there's still work to be done. What we started, I'd like to see things completed all the way through. It's becoming a little more difficult because I had to go back to my full‐time job… but it's very interesting, I think it's a different perspective, it's a refreshing process, the one in which I can work in concert with different professions coming together to make a difference. [PaCER intern]

Why am I with it? Basically when you do sit down with people who are your peers—in peer research—and you identify with them… I think we realize that we are very needed. Who is out there who is really doing this kind of research and is understanding them… by letting [decision makers] learn how to hear the experience of people? [PaCER intern]

The new competences may “recharge” patients’ confidence in voicing their knowledge as an equal partner:

[A year into PaCER training] when I was in hospital I felt like I had a pair of different glasses on. I feel that I have a different kind of awareness, the way I am seeing or analyzing things. I am pretty observant anyways, and I've always been an advocate for myself, but [PaCER experience] actually validates it even more, not in a bossy way, but it is important to speak up when you see something that's important to you… and ***be part of the team***.[PaCER intern]

#### 3.1.3 Conduit of under‐represented voices

In current health research, the patient is the data source. Research with PaCER brings a new analytical aspect to the patients’ input, which can enrich the decision‐making process:

Patient is absent from data analysis. They may be there for the collection because [researchers] need them as [research subjects], but the patient is missing from the input and the analysis phase. So I think if we provide that aspect to it, we will enrich the decision making process. [PaCER intern]

Participants saw opportunities for capturing authentic health experiences because patients are in a position to access patient‐centered data and interpret it in a unique way.

#### 3.1.4 Cocreating knowledge

PaCER methods, including specific approaches to focus group and interview facilitation, involve study participants in co‐creating research questions and co‐discovering the answers to those questions. According to their accounts, PaCER approaches generated peer‐to‐peer relationships that were particularly effective in allowing people to discover and co‐create knowledge:

Throughout the process, my own beliefs and experiences have undergone transformation as I have shared in others’ stories. They provided an incredible depth, illustrating the common experiences as well as individual variations. I am privileged to have been a part of this work. [PaCER intern]

This often involved sharing the experiences that had never been shared before, and participants in the internship studies discovered that their knowledge was both valued (as a contribution to valid research) and validated by peers (as an expression of understanding and support):

We are here to allow people to gain power through being part of the research… so it's really about them, not so much about us… this research has been designed this way. [PaCER intern]

### 3.2 Health care researchers: “deploy patient researchers as equal partners”

In the beginning, most of the health system boundary partners had hopeful expectations for this project, referring to the PaCER model's potential to increase patients’ education level, enhance their ability to manage their own health, encourage patients’ buy‐in into positive health behaviors, and convey the patient voice to inform health‐care delivery changes. Informants also expressed concerns about patient researchers’ possible bias or their potential judgemental stance. As the project progressed, the relationships shifted and the attitudes began to change, especially at the top decision‐making levels in AHS. As PaCER interns were invited to join the SCNs, these stakeholders noted the new perspectives.

#### 3.2.1 Initial expectations: The “cautiously optimistic” discourse

The concept of patients leading all aspects of the research process was disruptive because these new, unconventional roles taken by patient researchers departed from the traditional roles of a patient as a service user, volunteer, advisor or advocate.

I had no idea of what PaCER was about… I am familiar with research, and know about being a patient, and about engagement, but for me putting all three together was a major challenge—and it is a big challenge for the health‐care system. [Project team member]

For me this was kind of a foreign concept, because I am a health services researcher, and we hadn't engaged patients in this kind of ways before, and it led me to believe that this is a completely new science. [Health Scientist]

Patient engagement and leadership throughout the research process initially was perceived as odd, superfluous or even counterproductive. We heard cautions supported by the statements that academic researchers have skills and knowledge that are undoubtedly superior to those of the patients (even well‐trained patients); that patients might be too emotional about the issues of their health to be unbiased and objective; and that patients might be coming with the agenda of advocacy as opposed to a dispassionate position valued in research. We heard comments that patients would be certainly useful in constructing research questions, while the actual research would be better performed by experienced scientists. We heard a concern that resources might be better spent on enhancing patient engagement in some other, more traditional ways. Concerns were expressed about patient researchers’ possible inadequacy, preconceived position, lack of awareness of confidentiality and research ethics or their potential judgemental stance towards health‐care system.

In contrast, there was general consensus that patients would be able to ask research questions that traditional expert researchers would not necessarily think of, and that patients have the unique expertise that is currently under‐represented in research.

#### 3.2.2 Towards the partnership discourse

As the project progressed, we observed some remarkable changes in the relationship and language within the organizations and groups involved, but also encountered contextual, organizational and cultural barriers beyond our influence.

It's just so helpful to have a patient [a PaCER] right there in the middle, saying, ‘This is what patients feel, this is our experience in interviewing individuals,’ and this is a much larger voice at the table, so I find for me, it gets us on track much faster, building a better system. And it stops some of the arguing among the different disciplines about which is the direction we should go. The information and the perspective that they [PaCERs] bring is very different than the researcher or administrator or health care provider, and I find it often a much more balanced perspective than some of the rest of us have. [SCN executive]

Our collaborators among health‐care practitioners and leaders were beginning to notice how PaCER involvement influenced the general culture of their meetings and practices, as well as the language they routinely used. The following long vignette gives but one concrete example:

An example is… we spent a long time discussing, do we call people in the health system patients? clients? customers? and we've spent aeons just talking… and when [the PaCER] gets up and says, well, we want to be called patients, then I let go of that… And when a physician gets up and says, the patient's ‘medical home’ is the PCN, a physician's office, [the PaCER] gets up and says, ‘Well, my home is at [address]; what I want is the interface with the medical community, in a respectful way, and part of that is the physician's office. But I don't have a ‘medical home,’ like nobody owns me.’… And so for the language and the culture around that is really helpful when PaCERs articulate those messages for us… Those kinds of language are all important understandings. I think practitioners and other people could have said the same thing, ***but it wouldn*(*’*)*t have been heard to the same extent***…[SCN member]

#### 3.2.3 Reducing power imbalances

Even where tensions existed, participants experienced acceptance and respect from clinicians and other professional members of SCNs, which allowed PaCERs to provide valuable contribution to these groups and feel heard:

I would now find it difficult to work and not have them [PaCERs] in the room. It would be for me a real missing piece. And other people who haven't experienced that, they don't know what they are missing—they don't know what they don't have. [SCN member]

Having individuals at the [SCN] core committee level was very different this time [when PaCERs joined] … they [PaCERs] were so highly skilled representing larger groups, it was extremely helpful to have them there… I became more aware and realized why they were coming with the skill set that they have. In other arenas… where we had large groups meeting, I don't think we could have gotten to where we got to, without having the patients in the room. [AHS leader]

#### 3.2.4 The lessons of early engagement

PaCERs were invited to join the Bone and Joint SCN (BJSCN) Network at the launch of the SCNs in June 2012 when PaCER was in its early stages. PaCER engagement in the BJSCN, which had been planned as an end product of this project, started significantly ahead of schedule. We learned that cautions about the SCNs not being ready for the PaCERs, PaCER roles being “unclear or confusing,” or PaCERs not being prepared enough to contribute, were unfounded:

If you wait for the system to be ready, if you do your preliminary training and your preparation before, it will never happen. There is something about engagement that means, you ***engage***. And this is certainly one of the things we've learned—I have certainly learned a huge amount. Thank God we started this kind of work before our research project was over, because we wouldn't have understood what engagement with organizations mean. We have to keep finding new ways to engage. [Project team member]

As a result of this early engagement, the BJSCN remained a leader of uptake of PaCER model. The lesson we will take into the future is that our decision to step forward and begin early enabled growth and engagement in collaboration.

## 4 DISCUSSION

We began this initiative with the Outcome Mapping method of intentional design and data collection, adding grounded theory analysis to track emergent theoretical perspectives related to the changes within the culture of health care, which presented a way to study changing roles and relationships among patients, health researchers and health system planners. We encountered a few challenges and limitations. For example, as we expected, maintaining patient participation was challenging, mainly because of high intensity and expectations in training. These difficulties were not associated with age or education level. However, because all interns were patients, in a few cases health or family issues forced people to withdraw. As one of our participants noted, “You have the problem of engaging people without frightening them away with the enormity of the project.” Participants expressed concern about extending our research to marginalized communities. Although at least two of the five completed studies did reach out to some segments of these groups (eg, the aging immigrants and the homeless), there was a pressing need to focus on these patient populations in our future work.

Now that many organizations have been exposed to the new concept of PaCER, we have learned that in any given organizational or group context it takes time and a substantial effort in collaboration for the concept of PaCER to be truly understood, embraced and take root. The lesson we will take into the future is the need to be patient, clearly represent new roles, and to invite multiple partners to join. With only two years into the development of such a broad and new initiative by the time of this analysis, it was too early to expect any indications of a systemic impact on health‐care reform. However, we discovered several notable trends within relationships and culture, and also learned some significant lessons in this process.

The new PaCER model was well received by multiple stakeholders. In the current context of health care, patient engagement is considered essential, and PaCER provides a breakthrough by offering a vehicle for engaging patients and families in health decision making in a novel and meaningful way. We recognize that environmental factors might present a challenge to safeguarding the impact of this initiative. To seize the existing opportunities, we are building on our established partnerships and extend our collaboration to broader professional and academic groups. While our goal was to ensure that the voice of the patient was central to the change efforts underway within the SCNs, we also hoped that the concept of PaCER would have broader application within other sectors of AHS and health science. In this, it is clear that PaCER cuts across all three areas of engagement identified by Carman et al.^33^: direct patient care, organizational design and governance and policy making. That said, the most distinct potential impact of PaCER lies at the levels of influencing health culture and systems of care.^18^ Legitimate PaCER roles have potential to create a sustainable interface between patient‐produced health experience knowledge and policy making in health care.

## 5 CONCLUSIONS

As the initiation of the original project, PaCER has been embraced by many organizations in Alberta, including AHS, academic and community groups. The benefits of this initiative, albeit only emergent, have been demonstrated within two convergent streams of cultural and relationship change: (i) building the capacity of patients to engage confidently in a dialogue with clinicians and decision makers, and (ii) increasing the readiness for patient engagement uptake within targeted organizations. Patient researchers have begun to be welcomed by health‐care research and decision‐making organizations as “part of the team.” PaCER opens the door to a nascent science of health experience and engagement. We are now observing the first compelling indicators of success and we are left with many unanswered questions related to the long‐term impact of the fledgling new model. It is our priority to continue PaCER training and research to capture the long‐term impact, safeguard the positive change, and make PaCER accomplishments sustainable.

## CONFLICT OF INTEREST

DM is a Canada Research Chair in Health Systems and Services Research, and Arthur J.E. Child Chair in Rheumatology. SS and NM report grants from Canadian Foundation for Healthcare Improvement, grants from Alberta Health Services, and non‐financial support from Community Rehabilitation and Disability Studies Program (Community Health Sciences, Cumming School of Medicine, the University of Calgary), during the conduct of the study. TW declares no conflicts of interest.

## Note

1 In this article, we report the results of a 2‐year pilot initiative that was completed in 2013. This initiative laid the foundation for creating the PaCER Program that is now well established at the Cumming School of Medicine at the University of Calgary. For more information, visit our website at http://www.pacerinnovates.ca.

## References

[1] Swainston K , Summerbell C . The Effectiveness of Community Engagement Approaches and Methods for Health Promotion Interventions: Rapid Review. Middlesbrough, UK: University of Teesside; 2008.

[2] Gregory J . Conceptualising Consumer Engagement: A Review of the Literature. Melbourne, Australia: Australian Institute of Health Policy Studies; 2006.

[3] Gauvin F . Patient and Service User Engagement: An Environmental Scan. Ottawa, ON, Canada: Canadian Health Services Research Foundation; 2010.

[4] Coulter A , Parsons S , Askham J . Where are the patients in decision‐making about their own care? Policy Brief World Health Organization; 2008.

[5] National Institute for Health and Clinical Excellence . Community Engagement to Improve Health. London, UK: National Institute for Health and Clinical Excellence; 2008.

[6] Ferguson T . Consumer health informatics. Healthc Forum J. 1995;38:28‐33. 10154286

[7] Beresford P , Croft S . User Controlled Research: Scoping Review. London, UK: NIHR School for Social Care Research; 2012.

[8] National Institute for Health Research . INVOLVE. London, UK: National Institute for Health Research 2015.

[9] Patient‐Centered Outcome Research Institute . Washington, DC: Patient‐Centered Outcome Research Institute; 2014.

[10] King's College London . Service User Research Enterprise; 2014.

[11] Van der Geest S . Patients as co‐researchers? In: Fainzang SHH, Risor MB, eds. The Taste for Knowledge: Medical Anthropology Facing Medical Realities. Aarhus, Denmark: Aarhus University Press, 2010.

[12] Prager E . The older volunteer as research colleague: toward ‘generative participation’ for older adults. Educ Gerontol. 1995;21:209‐219.

[13] Marlett N . Partnership research in health promotion In: Thurston WESJ, Wiebe VJ, eds. Doing Health Promotion Research: The Science in Action. Calgary, AB: Health Promotions Research Group; 1998.

[14] Health Council of Canada . How Engaged are Canadians in Their Primary Care? Results from the 2010 Commonwealth Fund International Health Policy Survey. Toronto, On: Canadian Health Care Matters; 2011.

[15] Alberta Health Services . Clinical Networks: Terms of Reference. Edmonton, AB: Alberta Health Services; 2009.

[16] Alberta Health Services . Strategic Clinical Networks. A Primer & Working Document, Vol. 5 Edmonton, AB: Alberta Health Services; 2012.

[17] Alberta Health Services . Strategic Clinical Networks: Development Update. Edmonton, AB: Alberta Health Services; 2011.

[18] Marlett N , Shklarov S , Marshall D , Santana MJ , Wasylak T . Building new roles and relationships in research: a model of patient engagement research. Qual Life Res. 2015;24:1057‐1067. 25377348 10.1007/s11136-014-0845-y

[19] Wagner E . Chronic disease management: what will it take to improve care for chronic illness? Eff Clin Pract. 1998;1:2‐4. 10345255

[20] Coulter A , Ellins J . Effectiveness of strategies for informing, educating, and involving patients. BMJ. 2007;335:24‐27. 17615222 10.1136/bmj.39246.581169.80 PMC1910640

[21] Kreindler S . Patient involvement and the politics of methodology. Can Public Admin. 2009;52:113‐124.

[22] Gold SK , Abelson J , Charles CA . From rhetoric to reality: including patient voices in supportive cancer care planning. Health Expect. 2005;8:195‐209. 16098150 10.1111/j.1369-7625.2005.00334.x PMC5060302

[23] Earl S , Carden F , Smutylo T . Outcome Mapping: Building Learning and Reflection into Development Programs. Ottawa, ON: International Development Research Centre; 2001.

[24] Glaser BG . Basics of Grounded Theory Analysis: Emergence vs. Forcing. Mill Valley, CA: Sociology Press, 1992.

[25] Glaser BG . Theoretical Sensitivity: Advances in the Methodology of Grounded Theory. Mill Valley, CA: Sociology Press; 1978.

[26] Glaser BG , Strauss AL . The Discovery of Grounded Theory: Strategies for Qualitative Research. Chicago, IL: Aldine; 1967.

[27] Marlett N , Emes C . Grey Matters: A Guide to Collaborative Research with Seniors. Calgary, AB: University of Calgary Press; 2010.

[28] Elliot S , Eyles J , DeLuca J . Methodologies for community health assessment in areas of concern: a user‐friendly tool for policy and decision makers. Environ Health. 2001;109:817‐826. 10.1289/ehp.01109s6817 PMC1240617 11744500

[29] Monette D , Sullivan T , DeJong C . Applied Social Research: Tool for the Human Services, 8th edition. Belmont, CA: Brooks/Cole; 2011.

[30] Morgan D . Successful Focus Groups: Advancing the State of the Art. Newbury Park, CA: Sage; 1993.

[31] Rubin H , Rubin I . Qualitative Interviewing: The Art of Hearing Data. Thousand Oaks, CA: Sage; 1995.

[32] Holstein J , Gubrium J . The Active Interview. Thousand Oaks, CA: Sage; 1995.

[33] Carman KL , Dardess P , Maurer M , et al. Patient and family engagement: a framework for understanding the elements and developing interventions and policies. Health Aff. 2013;32:223‐231. 10.1377/hlthaff.2012.1133 23381514

