# Supplementary material for: “Part of the Team”: Mapping the outcomes of training patients for new roles in health research and planning
Source: Health Expect. 2017 Jun 28;20(6):1428–36. doi: 10.1111/hex.12591 (PMC5689226; doi:10.1111/hex.12591)
Supplement: Supplementary file 1 [file HEX-20-1428-s001.docx]

# Table S1. Patients Matter Intentional Design Framework

#### Excerpt from the Outcome Mapping Implementation Plan designed in November 2011

| **Patients Matter: Program Framework** |
| --- |
| **Vision:** Citizens living with osteoarthritis (OA) will sit on decision making committees and team conferences, together with clinicians, researchers, and policy makers within the formal health care system structures, as equal and knowledgeable partners in the discussions addressing population health and care delivery issues. Patients with OA will be empowered to recognize and take on their natural and acquired skills to join research teams within academic and clinical settings, to provide their expertise in the development, approval and evaluation of research grants, according to the principle *“nothing about us without us.”* The new role of a patient engagement researcher will be recognized and valued in both academic and health systems settings. No changes in health care delivery will happen without going through the meaningful, collaborative decision making process with service users. The category of service users who are becoming engaged will include not only people with OA who are already patients within the system, but also other citizens who are involved in promotion and prevention to answer meaningful questions to stay well. Ultimately, the engagement of service users will spread beyond the narrow area of OA. The value of patients’ engagement will be acknowledged within the AHS and Strategic Clinical Networks, as well as the academic settings, and patients’ contribution and expertise will be sought after by clinical and academic communities in Alberta. |
| **Mission:** In support of the vision, the project will serve as a catalyst of change by demonstrating the value of trained patient researchers engaged in AHS Strategic Clinical Network committees, beginning with a small group of patients engaged in the Bone and Joint Strategic Clinical Network. While promoting a small initiative, the project has an ultimate, broader mission to provide an example that will extend beyond the narrow disciplinary area in the future. The project will contribute to creating a new, commonly recognized domain of patient engagement research, and to promoting a new, specific role of patient engagement researcher. This will be achieved by teaching patient researchers in an inquiry-based way, so that they acquire very specific knowledge base and a particular set of skills in how to engage patients, how to capture and articulate their ideas, support these ideas with valid research, and bring them to the table. The project will work toward articulating what patient engagement research really is through implementing the training curriculum, research activities, and publishing patient engagement research. Through introducing and promoting in action the new role of patient engagement researcher, the project will target the culture of attitudes and relationships within two cultural domains: the academic research culture and the culture of organized health systems (including both clinicians and policy makers), beginning with the small local cluster of involved organizations. |

**Boundary Partners and Envisioned Outcomes**

| **Boundary Partner 1:**  Patient engagement researchers – project participants | **Outcome Challenge 1:** The program intends to see the group of project participants who are skilled and active as patient engagement researchers. They have mastered the specific research skills and know how to engage other patients, capture and articulate their ideas, support these ideas with valid research, and bring them to the table. Patient participants understand, value, and are able to act in their new roles as patient engagement researchers. They are becoming mentors to other patients who are interested to also take up these roles. They begin to participate in the Bone and Joint SCN as knowledgeable, competent and assertive partners in decision making. |
| --- | --- |
| **Boundary Partner 2:**  Bone and Joint Strategic Clinical Network, Alberta Health Services | **Outcome Challenge 2:** The program intends to see the Bone and Joint Strategic Clinical Network (BJSCN) that will deploy patient researchers as members on their working groups and committees as equal partners. In the longer term, the Alberta Health Services will adopt and use the model of patient engagement research in the BJSCN as a prototype for the development of the other Strategic Clinical Networks (for example, Addictions and Mental Health, Cardiac, or Cancer Networks). |
| **Boundary Partner 3:**  Institute for Public Health, Faculty of Medicine, U of C | **Outcome Challenge 3:** The program intends to see the Institute for Public Health that will sustain and support future patient engagement research opportunities through multidisciplinary faculty and students, as well as linkages with other boundary partners such as Alberta Health Services. |
