# Supplementary material for: “Part of the Team”: Mapping the outcomes of training patients for new roles in health research and planning
Source: Health Expect. 2017 Jun 28;20(6):1428–36. doi: 10.1111/hex.12591 (PMC5689226; doi:10.1111/hex.12591)
Supplement: Supplementary file 2 [file HEX-20-1428-s002.docx]

# Table S2. Patients Matter Evaluation Plan. Designed November 2011, updated throughout the duration of the project

| **Who will use the evaluation? How? When?** | **Questions** | **Information Sources** | **Evaluation Methods** | **Who will conduct and manage the evaluation?** | **Dates** |
| --- | --- | --- | --- | --- | --- |
| Project Team to:  1. Fulfil reporting requirements for funders  2. Present findings at conferences and relevant health services meetings and forums  3. Publish findings in a range academic and practice formats  4. Learn lessons from the project for future initiatives within academic, community and health services settings | How many attendees, trainees and volunteers participate in events?  How has the patients’ capacity change, in terms of their skills in patient engagement research?  How successful are patient engagement research projects in attempts at publication?  How many and what kinds of media appearances and academic presentations took place?  What strategies were most or least useful in increasing the patient researchers’ engagement capacity? | Progress markers and outcome journal  Project records, documentation, meeting notes  Interview transcripts, observation notes | Document review  Participant observation  Interviews with internal program team | Project coordinator, research team  Managed internally by Tracy Wasylak | Jan. 2012 to Sept. 2013 |
| **Who will use the evaluation? How? When?** | **Questions** | **Information Sources** | **Evaluation Methods** | **Who will conduct and manage the evaluation?** | **Dates** |
| Project Team to:  1. Fulfil reporting requirements for funders  2. Present findings at conferences and relevant health services meetings and forums  3. Publish findings in a range academic and practice formats  4. Learn lessons from the project for future initiatives within academic, community and health services settings | How effective is patient engagement research for increasing patient engagement within the boundary partner groups?  What are the changes in *patients’* willingness and confidence to be involved in improving health care?  What are the changes in the *collaborators’* willingness to accept patients as partners to be involved in improving health care?  What are the changes in patient engagement discourse within the boundary partners’ culture?  What are patients’ experiences of the project’s success and their own research processes? | Progress markers and outcome journal  Interview and focus groups transcripts  Observation notes  Project records, documentation, meeting notes (e.g., the number of factual events of patient involvement) | *Focus groups* with patient researchers in the beginning and by the end of the project  *Semi-structured interviews* with a sample of *patient researchers* and with a sample of *collaborators,* in the beginning and by the end of the project  Document review  Participant observation | Project coordinator, in collaboration with research team  Managed internally by Tracy Wasylak | Jan. 2012 to Sept. 2013 |
