# Supplementary material for: “Part of the Team”: Mapping the outcomes of training patients for new roles in health research and planning
Source: Health Expect. 2017 Jun 28;20(6):1428–36. doi: 10.1111/hex.12591 (PMC5689226; doi:10.1111/hex.12591)
Supplement: Supplementary file 3 [file HEX-20-1428-s003.docx]

# Data S1. Patient and Community Engagement Research (PACER) Model

Patient and Community Engagement Research Program (PACER) is part of the Institute for Public Health in the Faculty of Medicine at the University of Calgary. Academically, it is also part of the Community Rehabilitation and Disability Studies in the department of Community Health Sciences. The PACER mandate is to provide research development, supports and services to health researchers and teams, research foundations and Alberta Health Services and health care providers interested in adopting patient and community engagement methods as part of their research and planning processes.

PACER achieves this by teaching patients and caregivers to become skilled in engaging other patients, caregivers and communities in research and, upon graduation, making PACER graduates available to health care and research clients.

The PACER method has a distinct structure defined as ***set, collect, reflect*** (see the diagram below) to ensure that participants are meaningfully engaged throughout.

1. ***Set:*** This opening procedure, generally a focus group, invites representative patients and other relevant participants to become advisors and help *set* the stage for the study by refining the particular protocols (recruitment, locations, alliances), questions and data collection.
2. ***Collect:*** Particular techniques of data collection and analysis depend on the specifics of research questions and purposes of each given study.
3. ***Reflect:*** At this stage, participants from the initial *set* focus group (item 1) review findings and analysis with the PACER team and suggest knowledge utilization and recommendations for further research. This stage completes the circle of PACER procedures and prepares the data to be shared with the relevant health system.

**SET** and **REFLECT** focus groups are the hallmarks of the PACER method, serving to ensure a *meaningful patient involvement* and *contextual validity.*

**Reference:** Marlett, N. J., & Emes, C. (2010). *Grey Matters: A guide to collaborative research with seniors.* Calgary, AB: University of Calgary Press. Available through open access at <http://uofcpress.com/books/9781552382516>
